# 4-[(5-Hydr­oxy-3-methyl-1-phenyl-1*H*-pyrazol-4-yl)phenyl­meth­yl]-5-methyl-2-phenyl-1*H*-pyrazol-3(2*H*)-one ethanol hemisolvate

**DOI:** 10.1107/S1600536808039081

**Published:** 2008-12-10

**Authors:** Hoong-Kun Fun, Reza Kia, K. S. Girish, B. Kalluraya

**Affiliations:** a X-ray Crystallography Unit, School of Physics, Universiti Sains Malaysia, 11800 USM, Penang, Malaysia; b Department of Studies in Chemistry, Mangalore University, Mangalagangotri, Mangalore 574 199, India

## Abstract

The asymmetric unit of the title compound, C_27_H_24_N_4_O_2_·0.5C_2_H_6_O, comprises two crystallographically independent mol­ecules (*A* and *B*) with slightly different conformations, and one ethanol mol­ecule of crystallization. Intra­molecular C—H⋯O and O—H⋯O hydrogen bonds generate six- and eight-membered rings, producing *S*(6) and *S*(8) ring motifs, respectively. In mol­ecule *A*, one of the benzene rings is disordered over two positions, with site-occupancy factors of 0.542 (11) and 0.458 (11). The dihedral angles between the central benzene ring and the two outer benzene rings are 73.88 (9) and 82.6 (2)/88.9 (2)° in mol­ecule *A*, and 80.81 (8) and 79.38 (8)° in mol­ecule *B*. In the crystal structure, mol­ecules form infinite one-dimensional chains in the (101) plane. The crystal structure is stabilized by inter­molecular O—H⋯N, N—H⋯N, N—H⋯O and C—H⋯O hydrogen bonds, weak C—H⋯π and π–π [centroid–centroid = 3.5496 (1) Å] inter­actions.

## Related literature

For hydrogen-bond motifs, see: Bernstein *et al.* (1995 ▶). For bond-length data, see: Allen *et al.* (1987 ▶). For details of the biological activity of pyrazole derivatives, see: Burger & Iorio (1979 ▶, 1980 ▶); Kalluraya & Ramesh (2001 ▶); Holla *et al.* (1994 ▶); Windholz (2003 ▶).
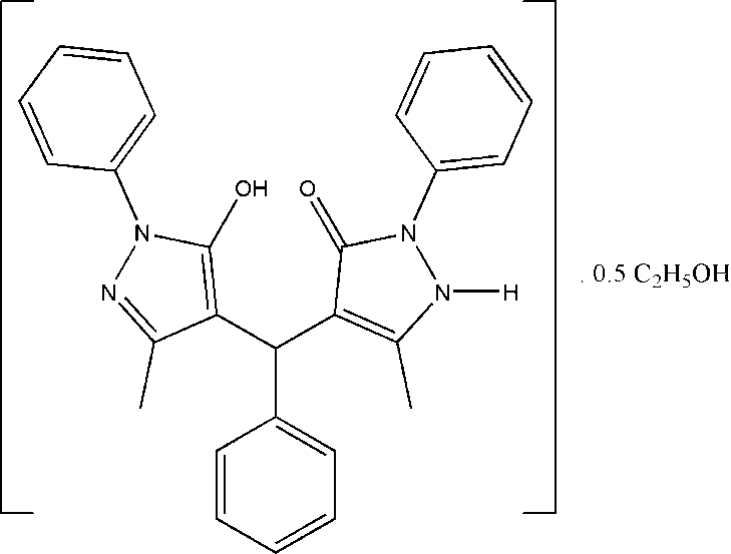


## Experimental

### 

#### Crystal data

C_27_H_24_N_4_O_2_·0.5C_2_H_6_O
*M* *_r_* = 459.54
Triclinic, 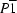

*a* = 8.3767 (2) Å
*b* = 13.9498 (3) Å
*c* = 20.4797 (4) Å
α = 101.074 (1)°
β = 93.723 (1)°
γ = 93.579 (1)°
*V* = 2336.74 (9) Å^3^
*Z* = 4
Mo *K*α radiation
μ = 0.09 mm^−1^
*T* = 100.0 (1) K
0.38 × 0.26 × 0.17 mm

#### Data collection

Bruker SMART APEXII CCD area-detector diffractometer
Absorption correction: multi-scan (**SADABS**; Bruker, 2005 ▶) *T*_min_ = 0.960, *T*_max_ = 0.986
41677 measured reflections
13462 independent reflections
9482 reflections with *I* > 2σ(*I*)
*R*_int_ = 0.043

#### Refinement

*R*[*F* ^2^ > 2σ(*F* ^2^)] = 0.055
*wR*(*F* ^2^) = 0.161
*S* = 1.03
13462 reflections
632 parameters
42 restraints
H atoms treated by a mixture of independent and constrained refinement
Δρ_max_ = 0.40 e Å^−3^
Δρ_min_ = −0.43 e Å^−3^

### 

Data collection: *APEX2* (Bruker, 2005 ▶); cell refinement: *APEX2*; data reduction: *SAINT* (Bruker, 2005 ▶); program(s) used to solve structure: *SHELXTL* (Sheldrick, 2008 ▶); program(s) used to refine structure: *SHELXTL*; molecular graphics: *SHELXTL*; software used to prepare material for publication: *SHELXTL* and *PLATON* (Spek, 2003 ▶).

## Supplementary Material

Crystal structure: contains datablocks global, I. DOI: 10.1107/S1600536808039081/sj2553sup1.cif

Structure factors: contains datablocks I. DOI: 10.1107/S1600536808039081/sj2553Isup2.hkl

Additional supplementary materials:  crystallographic information; 3D view; checkCIF report

## Figures and Tables

**Table 1:** Hydrogen-bond geometry (Å, °)

*D*—H⋯*A*	*D*—H	H⋯*A*	*D*⋯*A*	*D*—H⋯*A*
O2*A*—H2*OA*⋯O1*A*	0.82	1.76	2.5755 (16)	173
O3—H3⋯N3*A*^i^	0.82	2.11	2.923 (2)	173
O2*B*—H2*OB*⋯O1*B*	0.82	1.68	2.4970 (16)	176
N2*A*—H2*NA*⋯N3*B*^ii^	0.93 (3)	1.88 (3)	2.810 (2)	176 (2)
N2*B*—H2*NB*⋯O1*A*^iii^	0.95 (2)	1.75 (2)	2.6922 (19)	173 (2)
C1*B*—H1*BA*⋯O1*B*	0.93	2.37	2.925 (2)	118
C2*B*—H2*BA*⋯O3^iv^	0.93	2.48	3.313 (2)	149
C21*A*—H21*A*⋯O2*A*	0.93	2.41	2.910 (5)	114
C5*B*—H5*BA*⋯*Cg*1^i^	0.93	2.89	3.595 (3)	134
C29—H29*B*⋯*Cg*2^iv^	0.97	2.80	3.492 (2)	129

Symmetry codes: (i) 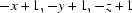; (ii) 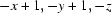; (iii) 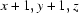; (iv) 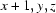. *Cg*1 and *Cg*2 are the centroids of the C11*A*–C16*A* and C20*C*–C25*C* benzene rings, respectively.

**Fig. 1.**
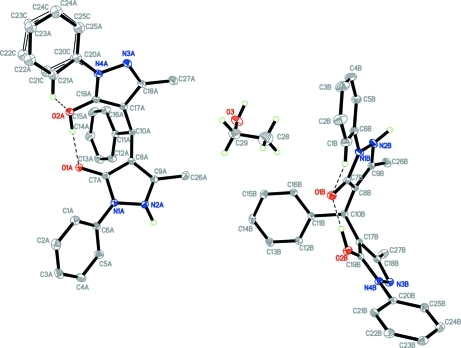
The molecular structure of (I) with atom labels and 40% probability ellipsoids for non-H atoms. For clarity, only the hydrogen atoms involved in the hydrogen bonding and the hydrogen of the ethanol molecule are shown. Intramolecular interactions are shown as dashed lines. The solid bonds show the major disorder component.

**Fig. 2.**
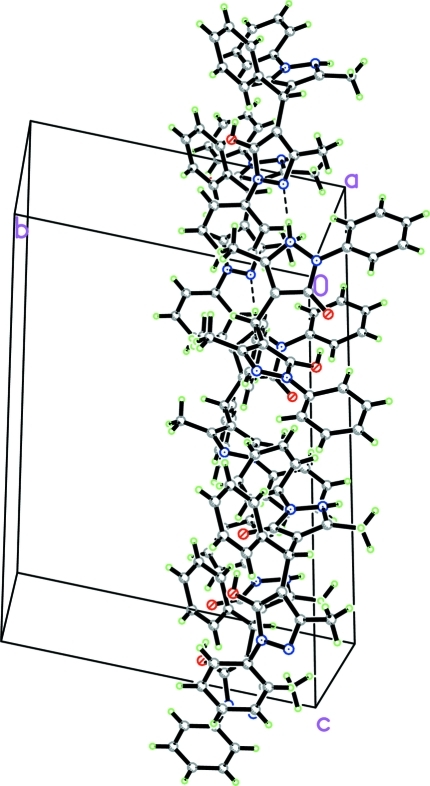
The crystal packing of the major disorder component of (I), showing the infinite 1-dimensional chains along (1 0 1). Intermolecular interactions are shown as dashed lines.

## supplementary crystallographic information

### Comment

Pyrazole derivatives are reported to possess varied biological activities such as anti-inflammatory (Windholz 2003), analgesic (Windholz 2003), hypoglysemic, seditive (Burger *et al.*, 1979), hypnotic (Burger *et al.*, 1980), antifungal and antibacterial (Kalluraya *et al.*, 2001) activities. Propenones are also found to show good antibacterial activity (Holla *et al.* 1994). Prompted by these observations, we planned to synthesize propenones containing pyrazole moiety and we report here the structure of the title compound, I, Fig. 1.

The bond lengths (Allen *et al.*, 1987) and angles in the title compound have normal values. The asymmetric unit of the title compound, is composed of two crystallographically independent molecules (A and B) with slightly different conformations and one ethanol molecule of crystallization. Intramolecular C—H···O and O—H···O hydrogen bonds generate six- and eight-membered rings, producing *S*(6) and *S*(8) ring motifs (Bernstein *et al.* 1995), respectively. In molecule A, one of the benzene rings is disordered over two positions with the site-occupancy factors 0.542 (11)/0.458 (11). The dihedral angles between the central benzene ring and the two outer benzene rings are 73.88 (9) and 82.6 (2)/88.9 (2)° in molecule A; and 80.81 (8) and 79.38 (8)° in molecule B, respectively. In the crystal structure, molecules are linked into infinite 1-dimensional chains in the (101)-plane (Fig. 2). The crystal structure is stabilized by intermolecular O—H···N, N—H···N, N—H···O, and C—H···O hydrogen bonds, weak C—H···π (Table 1, *Cg*1 and *Cg*2 are the centroids of the C11A–C16A and C20C–C25C benzene rings respectively) and π-π interactions [*Cg3*···*Cg4* = 3.5496 (10) Å; symmetry code: -1 + *x*,-1 + *y*, *z*; *Cg*3 and *Cg*4 are the centroids of the C1A–C6A and N1B/N2B/C7B–C9B benzene rings respectively].

### Experimental

The title compound was prepared by the direct fusion of 1-phenyl- 3-methyl 5-pyrazolone (0.1 mole) with benzaldehyde (0.1 mole) at 413 K for 3 h. The reaction mixture was cooled to room temperature and stirred with methanol using a glass rod. The mixture was then filtered to obtain a solid product. Single crystals suitable for X-ray analysis were obtained by recrystallization from ethanol under slow evaporation (m.p. 351–353 K).

### Refinement

N-bonded H atoms were located from the difference Fourier map and freely refined. H atoms of the hydroxy groups were positioned with freely rotating O—H bonds and constrained with a fixed distance of 0.82 Å. Other hydrogen atoms were positioned geometrically and refined using a riding model with C—H = 0.93–0.98 Å and *U*_iso_(H) = 1.2 or 1.5 *U*_eq_(C). A rotating-group model was applied to the methyl hydrogen atoms. One of the benzene rings in molecule A is disordered over two positions with site-occupancy factors 0.542 (11)/0.458 (11). The disorder was modeled based on a rigid bond model and the C atoms refined isotropically.

### Crystal data

**Table:**

C_27_H_24_N_4_O_2_·0.5C_2_H_6_O	*Z* = 4
*M**_r_* = 459.54	*F*(000) = 972
Triclinic, *P*1	*D*_x_ = 1.306 Mg m^−^^3^
Hall symbol: -P 1	Melting point: 352 K
*a* = 8.3767 (2) Å	Mo *K*α radiation, λ = 0.71073 Å
*b* = 13.9498 (3) Å	Cell parameters from 8872 reflections
*c* = 20.4797 (4) Å	θ = 2.5–31.5°
α = 101.074 (1)°	µ = 0.09 mm^−^^1^
β = 93.723 (1)°	*T* = 100 K
γ = 93.579 (1)°	Block, yellow
*V* = 2336.74 (9) Å^3^	0.38 × 0.26 × 0.17 mm

### Data collection

**Table:**

Bruker SMART APEXII CCD area-detector diffractometer	13462 independent reflections
Radiation source: fine-focus sealed tube	9482 reflections with *I* > 2σ(*I*)
graphite	*R*_int_ = 0.043
φ and ω scans	θ_max_ = 30.0°, θ_min_ = 1.5°
Absorption correction: multi-scan (*SADABS*; Bruker, 2005)	*h* = −11→11
*T*_min_ = 0.960, *T*_max_ = 0.986	*k* = −19→19
41677 measured reflections	*l* = −26→28

### Refinement

**Table:**

Refinement on *F*^2^	Primary atom site location: structure-invariant direct methods
Least-squares matrix: full	Secondary atom site location: difference Fourier map
*R*[*F*^2^ > 2σ(*F*^2^)] = 0.055	Hydrogen site location: inferred from neighbouring sites
*wR*(*F*^2^) = 0.161	H atoms treated by a mixture of independent and constrained refinement
*S* = 1.03	*w* = 1/[σ^2^(*F*_o_^2^) + (0.0833*P*)^2^ + 0.5342*P*] where *P* = (*F*_o_^2^ + 2*F*_c_^2^)/3
13462 reflections	(Δ/σ)_max_ < 0.001
632 parameters	Δρ_max_ = 0.40 e Å^−^^3^
42 restraints	Δρ_min_ = −0.43 e Å^−^^3^

### Special details

**Table:**

Experimental. The low-temperature data was collected with the Oxford Cyrosystem Cobra low-temperature attachment.
Geometry. All e.s.d.'s (except the e.s.d. in the dihedral angle between two l.s. planes) are estimated using the full covariance matrix. The cell e.s.d.'s are taken into account individually in the estimation of e.s.d.'s in distances, angles and torsion angles; correlations between e.s.d.'s in cell parameters are only used when they are defined by crystal symmetry. An approximate (isotropic) treatment of cell e.s.d.'s is used for estimating e.s.d.'s involving l.s. planes.
Refinement. Refinement of *F*^2^ against ALL reflections. The weighted *R*-factor *wR* and goodness of fit *S* are based on *F*^2^, conventional *R*-factors *R* are based on *F*, with *F* set to zero for negative *F*^2^. The threshold expression of *F*^2^ > σ(*F*^2^) is used only for calculating *R*-factors(gt) *etc*. and is not relevant to the choice of reflections for refinement. *R*-factors based on *F*^2^ are statistically about twice as large as those based on *F*, and *R*- factors based on ALL data will be even larger.

### Fractional atomic coordinates and isotropic or equivalent isotropic displacement parameters (Å^2^)

**Table:**

	*x*	*y*	*z*	*U*_iso_*/*U*_eq_	Occ. (<1)
O1A	0.04693 (14)	0.06678 (8)	0.31199 (5)	0.0191 (2)	
O2A	0.06851 (15)	0.10931 (9)	0.44047 (6)	0.0228 (3)	
H2OA	0.0534	0.0942	0.3997	0.034*	
N1A	0.15609 (16)	0.11331 (10)	0.22035 (6)	0.0160 (3)	
N2A	0.21144 (16)	0.20043 (10)	0.20320 (7)	0.0164 (3)	
N3A	0.30731 (16)	0.31761 (10)	0.52500 (7)	0.0186 (3)	
N4A	0.24860 (16)	0.22036 (10)	0.51385 (7)	0.0173 (3)	
C20A	0.3120 (10)	0.1609 (6)	0.5553 (5)	0.021 (2)*	0.542 (11)
C21A	0.3284 (6)	0.0608 (3)	0.5268 (2)	0.0232 (10)*	0.542 (11)
H21A	0.2984	0.0369	0.4818	0.028*	0.542 (11)
C22A	0.3892 (7)	−0.0009 (3)	0.5663 (2)	0.0322 (11)*	0.542 (11)
H22A	0.3934	−0.0670	0.5480	0.039*	0.542 (11)
C23A	0.4433 (7)	0.0336 (4)	0.6317 (3)	0.0304 (13)*	0.542 (11)
H23A	0.4779	−0.0089	0.6586	0.036*	0.542 (11)
C24A	0.4451 (8)	0.1300 (4)	0.6562 (3)	0.0352 (16)*	0.542 (11)
H24A	0.4884	0.1552	0.6995	0.042*	0.542 (11)
C25A	0.3826 (7)	0.1931 (4)	0.6171 (3)	0.0305 (16)*	0.542 (11)
H25A	0.3903	0.2600	0.6347	0.037*	0.542 (11)
C20C	0.3025 (11)	0.1530 (7)	0.5557 (5)	0.016 (2)*	0.458 (11)
C21C	0.2784 (8)	0.0544 (4)	0.5377 (3)	0.0301 (13)*	0.458 (11)
H21C	0.2226	0.0262	0.4971	0.036*	0.458 (11)
C22C	0.3367 (8)	−0.0048 (3)	0.5795 (3)	0.0282 (13)*	0.458 (11)
H22C	0.3235	−0.0726	0.5663	0.034*	0.458 (11)
C23C	0.4148 (7)	0.0373 (4)	0.6411 (3)	0.0186 (11)*	0.458 (11)
H23C	0.4637	−0.0013	0.6676	0.022*	0.458 (11)
C24C	0.4187 (7)	0.1381 (4)	0.6624 (3)	0.0146 (11)*	0.458 (11)
H24C	0.4645	0.1660	0.7049	0.018*	0.458 (11)
C25C	0.3572 (7)	0.1980 (4)	0.6228 (3)	0.0134 (12)*	0.458 (11)
H25C	0.3514	0.2646	0.6387	0.016*	0.458 (11)
C1A	0.25204 (19)	−0.05002 (12)	0.20784 (9)	0.0205 (3)	
H1AA	0.2910	−0.0362	0.2525	0.025*	
C2A	0.2680 (2)	−0.14120 (13)	0.16831 (10)	0.0252 (4)	
H2AA	0.3177	−0.1886	0.1868	0.030*	
C3A	0.2111 (2)	−0.16232 (13)	0.10179 (9)	0.0247 (4)	
H3AA	0.2225	−0.2235	0.0757	0.030*	
C4A	0.1370 (2)	−0.09156 (13)	0.07439 (9)	0.0259 (4)	
H4AA	0.0992	−0.1053	0.0296	0.031*	
C5A	0.1188 (2)	−0.00035 (13)	0.11323 (8)	0.0218 (3)	
H5AA	0.0679	0.0467	0.0948	0.026*	
C6A	0.17717 (18)	0.02021 (12)	0.17991 (8)	0.0159 (3)	
C7A	0.09891 (18)	0.13337 (12)	0.28245 (7)	0.0150 (3)	
C8A	0.11043 (17)	0.23705 (11)	0.30291 (7)	0.0142 (3)	
C9A	0.17638 (18)	0.27454 (12)	0.25245 (8)	0.0156 (3)	
C10A	0.04788 (18)	0.29225 (12)	0.36615 (7)	0.0147 (3)	
H10A	0.0638	0.3619	0.3643	0.018*	
C11A	−0.13429 (18)	0.27032 (11)	0.36632 (8)	0.0150 (3)	
C12A	−0.23042 (19)	0.24023 (13)	0.30674 (8)	0.0200 (3)	
H12A	−0.1830	0.2303	0.2664	0.024*	
C13A	−0.3961 (2)	0.22486 (14)	0.30669 (9)	0.0242 (4)	
H13A	−0.4583	0.2041	0.2666	0.029*	
C14A	−0.4685 (2)	0.24037 (14)	0.36616 (9)	0.0253 (4)	
H14A	−0.5792	0.2301	0.3663	0.030*	
C15A	−0.3747 (2)	0.27133 (15)	0.42537 (9)	0.0258 (4)	
H15A	−0.4231	0.2827	0.4654	0.031*	
C16A	−0.2086 (2)	0.28570 (13)	0.42591 (8)	0.0209 (3)	
H16A	−0.1471	0.3057	0.4663	0.025*	
C17A	0.14073 (18)	0.28029 (12)	0.42951 (7)	0.0153 (3)	
C18A	0.24186 (18)	0.35227 (12)	0.47389 (8)	0.0170 (3)	
C19A	0.14672 (19)	0.19775 (12)	0.45748 (8)	0.0168 (3)	
C26A	0.2124 (2)	0.37793 (13)	0.24580 (9)	0.0249 (4)	
H26A	0.2791	0.3795	0.2096	0.037*	
H26B	0.2673	0.4143	0.2865	0.037*	
H26C	0.1140	0.4067	0.2371	0.037*	
C27A	0.2781 (2)	0.45637 (13)	0.46862 (9)	0.0262 (4)	
H27A	0.3384	0.4908	0.5086	0.039*	
H27B	0.1795	0.4865	0.4629	0.039*	
H27C	0.3397	0.4588	0.4310	0.039*	
O2B	0.93942 (14)	0.63369 (8)	0.04138 (6)	0.0179 (2)	
H2OB	0.9272	0.6440	0.0815	0.027*	
O1B	0.91354 (13)	0.66225 (8)	0.16426 (5)	0.0182 (2)	
N4B	0.90424 (15)	0.71344 (10)	−0.04759 (6)	0.0150 (3)	
N3B	0.81223 (16)	0.78239 (10)	−0.06849 (7)	0.0170 (3)	
N2B	0.88285 (17)	0.89755 (11)	0.25148 (7)	0.0190 (3)	
N1B	0.94837 (16)	0.80809 (10)	0.24321 (7)	0.0173 (3)	
C1B	1.1437 (2)	0.70528 (14)	0.28164 (9)	0.0243 (4)	
H1BA	1.1468	0.6710	0.2381	0.029*	
C2B	1.2358 (2)	0.68007 (16)	0.33339 (9)	0.0314 (4)	
H2BA	1.3002	0.6278	0.3247	0.038*	
C3B	1.2329 (2)	0.73225 (17)	0.39840 (9)	0.0339 (5)	
H3BA	1.2954	0.7149	0.4329	0.041*	
C4B	1.1377 (2)	0.80951 (16)	0.41180 (9)	0.0306 (4)	
H4BA	1.1367	0.8444	0.4553	0.037*	
C5B	1.0431 (2)	0.83566 (14)	0.36065 (8)	0.0233 (4)	
H5BA	0.9785	0.8877	0.3696	0.028*	
C6B	1.04641 (19)	0.78271 (13)	0.29578 (8)	0.0196 (3)	
C7B	0.87984 (18)	0.74959 (12)	0.18494 (8)	0.0157 (3)	
C8B	0.76814 (18)	0.80655 (11)	0.15665 (7)	0.0147 (3)	
C9B	0.77990 (19)	0.89732 (12)	0.19769 (8)	0.0172 (3)	
C10B	0.65481 (18)	0.76875 (11)	0.09613 (7)	0.0142 (3)	
H10B	0.5828	0.8209	0.0936	0.017*	
C11B	0.54597 (17)	0.67919 (11)	0.10342 (8)	0.0137 (3)	
C12B	0.45406 (19)	0.62474 (12)	0.04803 (8)	0.0186 (3)	
H12B	0.4646	0.6404	0.0063	0.022*	
C13B	0.3471 (2)	0.54760 (13)	0.05414 (9)	0.0227 (3)	
H13B	0.2858	0.5125	0.0167	0.027*	
C14B	0.3314 (2)	0.52257 (13)	0.11590 (9)	0.0217 (3)	
H14B	0.2603	0.4704	0.1199	0.026*	
C15B	0.42219 (19)	0.57569 (12)	0.17175 (8)	0.0190 (3)	
H15B	0.4123	0.5591	0.2132	0.023*	
C16B	0.52806 (18)	0.65393 (12)	0.16558 (8)	0.0163 (3)	
H16B	0.5876	0.6898	0.2032	0.020*	
C17B	0.73824 (18)	0.75532 (11)	0.03188 (8)	0.0142 (3)	
C18B	0.71295 (19)	0.80612 (12)	−0.02041 (8)	0.0160 (3)	
C19B	0.86207 (18)	0.69663 (11)	0.01323 (7)	0.0140 (3)	
C20B	1.04430 (18)	0.68613 (12)	−0.08021 (7)	0.0154 (3)	
C21B	1.09192 (19)	0.59116 (12)	−0.08653 (8)	0.0183 (3)	
H21B	1.0316	0.5441	−0.0701	0.022*	
C22B	1.2306 (2)	0.56752 (13)	−0.11770 (8)	0.0216 (3)	
H22B	1.2633	0.5043	−0.1220	0.026*	
C23B	1.3209 (2)	0.63729 (13)	−0.14245 (8)	0.0221 (3)	
H23B	1.4139	0.6208	−0.1631	0.027*	
C24B	1.2724 (2)	0.73159 (13)	−0.13640 (8)	0.0211 (3)	
H24B	1.3327	0.7783	−0.1531	0.025*	
C25B	1.13422 (19)	0.75638 (12)	−0.10552 (8)	0.0190 (3)	
H25B	1.1015	0.8196	−0.1017	0.023*	
C26B	0.7042 (2)	0.98896 (12)	0.19062 (9)	0.0231 (4)	
H26D	0.7049	1.0310	0.2337	0.035*	
H26E	0.5955	0.9730	0.1719	0.035*	
H26F	0.7633	1.0219	0.1617	0.035*	
C27B	0.5924 (2)	0.87954 (13)	−0.02620 (9)	0.0232 (4)	
H27D	0.5940	0.8972	−0.0692	0.035*	
H27E	0.6185	0.9369	0.0080	0.035*	
H27F	0.4873	0.8516	−0.0211	0.035*	
C28	0.8096 (3)	0.54578 (19)	0.31209 (13)	0.0480 (6)	
H28A	0.8887	0.5014	0.2970	0.072*	
H28B	0.8553	0.5938	0.3498	0.072*	
H28C	0.7754	0.5780	0.2768	0.072*	
C29	0.6683 (3)	0.48982 (15)	0.33204 (10)	0.0331 (4)	
H29A	0.6245	0.4404	0.2940	0.040*	
H29B	0.7043	0.4563	0.3670	0.040*	
O3	0.54565 (15)	0.54997 (10)	0.35503 (7)	0.0291 (3)	
H3	0.5803	0.5897	0.3886	0.044*	
H2NA	0.208 (3)	0.2048 (18)	0.1585 (13)	0.048 (7)*	
H2NB	0.944 (3)	0.9543 (17)	0.2753 (11)	0.035 (6)*	

### Atomic displacement parameters (Å^2^)

**Table:**

	*U*^11^	*U*^22^	*U*^33^	*U*^12^	*U*^13^	*U*^23^
O1A	0.0252 (6)	0.0155 (6)	0.0156 (5)	−0.0042 (5)	0.0035 (5)	0.0021 (4)
O2A	0.0316 (7)	0.0195 (6)	0.0155 (5)	−0.0062 (5)	−0.0026 (5)	0.0028 (5)
N1A	0.0195 (6)	0.0140 (6)	0.0144 (6)	0.0000 (5)	0.0034 (5)	0.0020 (5)
N2A	0.0203 (6)	0.0152 (7)	0.0145 (6)	0.0016 (5)	0.0046 (5)	0.0035 (5)
N3A	0.0175 (6)	0.0184 (7)	0.0180 (6)	−0.0009 (5)	−0.0008 (5)	0.0005 (5)
N4A	0.0181 (6)	0.0175 (7)	0.0152 (6)	0.0011 (5)	−0.0005 (5)	0.0011 (5)
C1A	0.0178 (7)	0.0225 (9)	0.0215 (8)	0.0052 (7)	0.0031 (6)	0.0037 (7)
C2A	0.0239 (8)	0.0207 (9)	0.0336 (10)	0.0092 (7)	0.0087 (7)	0.0073 (7)
C3A	0.0228 (8)	0.0182 (9)	0.0319 (9)	0.0005 (7)	0.0112 (7)	−0.0011 (7)
C4A	0.0301 (9)	0.0243 (9)	0.0203 (8)	0.0011 (8)	0.0024 (7)	−0.0029 (7)
C5A	0.0259 (8)	0.0190 (8)	0.0202 (8)	0.0044 (7)	0.0007 (7)	0.0028 (7)
C6A	0.0150 (7)	0.0153 (8)	0.0169 (7)	0.0017 (6)	0.0044 (6)	0.0005 (6)
C7A	0.0125 (6)	0.0186 (8)	0.0132 (7)	0.0001 (6)	0.0007 (5)	0.0015 (6)
C8A	0.0116 (6)	0.0163 (8)	0.0140 (7)	−0.0004 (6)	0.0008 (5)	0.0018 (6)
C9A	0.0145 (7)	0.0175 (8)	0.0146 (7)	0.0018 (6)	0.0004 (6)	0.0024 (6)
C10A	0.0149 (7)	0.0146 (7)	0.0140 (7)	0.0003 (6)	0.0023 (6)	0.0012 (6)
C11A	0.0140 (7)	0.0150 (7)	0.0163 (7)	0.0032 (6)	0.0013 (6)	0.0029 (6)
C12A	0.0173 (7)	0.0241 (9)	0.0172 (7)	0.0036 (7)	0.0013 (6)	−0.0001 (6)
C13A	0.0159 (7)	0.0292 (10)	0.0252 (9)	0.0031 (7)	−0.0030 (7)	0.0004 (7)
C14A	0.0134 (7)	0.0343 (10)	0.0304 (9)	0.0041 (7)	0.0031 (7)	0.0103 (8)
C15A	0.0172 (8)	0.0416 (11)	0.0222 (8)	0.0082 (8)	0.0068 (7)	0.0119 (8)
C16A	0.0177 (7)	0.0298 (9)	0.0164 (7)	0.0057 (7)	0.0024 (6)	0.0057 (7)
C17A	0.0143 (7)	0.0178 (8)	0.0127 (7)	0.0009 (6)	0.0023 (6)	0.0003 (6)
C18A	0.0144 (7)	0.0197 (8)	0.0152 (7)	0.0006 (6)	0.0011 (6)	−0.0008 (6)
C19A	0.0175 (7)	0.0182 (8)	0.0132 (7)	−0.0004 (6)	0.0017 (6)	−0.0002 (6)
C26A	0.0349 (10)	0.0205 (9)	0.0203 (8)	−0.0006 (7)	0.0078 (7)	0.0051 (7)
C27A	0.0297 (9)	0.0231 (9)	0.0232 (8)	−0.0042 (7)	−0.0053 (7)	0.0024 (7)
O2B	0.0235 (6)	0.0172 (6)	0.0145 (5)	0.0064 (5)	0.0033 (5)	0.0046 (4)
O1B	0.0213 (6)	0.0160 (6)	0.0165 (5)	0.0032 (5)	−0.0001 (4)	0.0013 (4)
N4B	0.0165 (6)	0.0145 (6)	0.0146 (6)	0.0041 (5)	0.0031 (5)	0.0030 (5)
N3B	0.0188 (6)	0.0170 (7)	0.0163 (6)	0.0050 (5)	0.0023 (5)	0.0048 (5)
N2B	0.0200 (7)	0.0140 (7)	0.0202 (7)	−0.0016 (5)	0.0006 (6)	−0.0026 (5)
N1B	0.0176 (6)	0.0163 (7)	0.0160 (6)	−0.0006 (5)	−0.0005 (5)	−0.0008 (5)
C1B	0.0206 (8)	0.0336 (10)	0.0181 (8)	0.0023 (7)	0.0009 (6)	0.0038 (7)
C2B	0.0243 (9)	0.0463 (12)	0.0251 (9)	0.0097 (9)	0.0001 (7)	0.0089 (9)
C3B	0.0262 (9)	0.0558 (14)	0.0207 (9)	0.0038 (9)	−0.0029 (7)	0.0116 (9)
C4B	0.0295 (9)	0.0453 (12)	0.0144 (8)	−0.0051 (9)	0.0007 (7)	0.0020 (8)
C5B	0.0227 (8)	0.0281 (9)	0.0172 (8)	−0.0033 (7)	0.0026 (7)	0.0010 (7)
C6B	0.0158 (7)	0.0265 (9)	0.0158 (7)	−0.0043 (6)	0.0001 (6)	0.0049 (7)
C7B	0.0163 (7)	0.0164 (8)	0.0133 (7)	−0.0023 (6)	0.0025 (6)	0.0005 (6)
C8B	0.0157 (7)	0.0147 (7)	0.0130 (7)	−0.0008 (6)	0.0027 (6)	0.0009 (6)
C9B	0.0157 (7)	0.0177 (8)	0.0176 (7)	−0.0009 (6)	0.0050 (6)	0.0018 (6)
C10B	0.0147 (7)	0.0141 (7)	0.0139 (7)	0.0022 (6)	0.0024 (6)	0.0016 (6)
C11B	0.0116 (6)	0.0128 (7)	0.0164 (7)	0.0023 (6)	0.0025 (5)	0.0010 (6)
C12B	0.0198 (7)	0.0189 (8)	0.0165 (7)	−0.0008 (6)	−0.0003 (6)	0.0037 (6)
C13B	0.0201 (8)	0.0226 (9)	0.0231 (8)	−0.0047 (7)	−0.0045 (7)	0.0026 (7)
C14B	0.0176 (7)	0.0195 (9)	0.0282 (9)	−0.0039 (6)	0.0003 (7)	0.0072 (7)
C15B	0.0184 (7)	0.0205 (8)	0.0190 (8)	0.0022 (6)	0.0042 (6)	0.0053 (6)
C16B	0.0160 (7)	0.0168 (8)	0.0154 (7)	0.0021 (6)	0.0020 (6)	0.0010 (6)
C17B	0.0134 (6)	0.0131 (7)	0.0158 (7)	0.0002 (6)	0.0008 (6)	0.0025 (6)
C18B	0.0165 (7)	0.0151 (8)	0.0160 (7)	0.0007 (6)	0.0007 (6)	0.0025 (6)
C19B	0.0153 (7)	0.0124 (7)	0.0134 (7)	−0.0011 (6)	0.0011 (6)	0.0009 (6)
C20B	0.0157 (7)	0.0177 (8)	0.0120 (7)	0.0021 (6)	0.0006 (6)	0.0009 (6)
C21B	0.0193 (7)	0.0160 (8)	0.0185 (7)	0.0009 (6)	0.0015 (6)	0.0006 (6)
C22B	0.0212 (8)	0.0220 (9)	0.0220 (8)	0.0075 (7)	0.0036 (7)	0.0024 (7)
C23B	0.0190 (8)	0.0286 (9)	0.0186 (8)	0.0050 (7)	0.0036 (6)	0.0024 (7)
C24B	0.0192 (8)	0.0255 (9)	0.0197 (8)	0.0007 (7)	0.0032 (6)	0.0072 (7)
C25B	0.0199 (7)	0.0174 (8)	0.0198 (8)	0.0026 (6)	0.0017 (6)	0.0036 (6)
C26B	0.0252 (8)	0.0162 (8)	0.0262 (9)	0.0015 (7)	0.0026 (7)	−0.0006 (7)
C27B	0.0229 (8)	0.0281 (9)	0.0216 (8)	0.0107 (7)	0.0034 (7)	0.0088 (7)
C28	0.0409 (12)	0.0560 (16)	0.0466 (13)	0.0062 (11)	0.0164 (11)	0.0035 (12)
C29	0.0431 (11)	0.0272 (10)	0.0292 (10)	0.0105 (9)	0.0053 (9)	0.0025 (8)
O3	0.0254 (6)	0.0281 (7)	0.0295 (7)	0.0029 (6)	−0.0028 (5)	−0.0038 (6)

### Geometric parameters (Å, °)

**Table:**

O1A—C7A	1.2720 (19)	C27A—H27B	0.9600
O2A—C19A	1.333 (2)	C27A—H27C	0.9600
O2A—H2OA	0.8200	O2B—C19B	1.3191 (18)
N1A—C7A	1.3727 (19)	O2B—H2OB	0.8200
N1A—N2A	1.3892 (18)	O1B—C7B	1.2664 (19)
N1A—C6A	1.428 (2)	N4B—C19B	1.3749 (19)
N2A—C9A	1.359 (2)	N4B—N3B	1.3821 (17)
N2A—H2NA	0.93 (3)	N4B—C20B	1.4240 (19)
N3A—C18A	1.335 (2)	N3B—C18B	1.3366 (19)
N3A—N4A	1.3848 (19)	N2B—C9B	1.354 (2)
N4A—C19A	1.363 (2)	N2B—N1B	1.3798 (19)
N4A—C20A	1.400 (9)	N2B—H2NB	0.95 (2)
N4A—C20C	1.461 (10)	N1B—C7B	1.378 (2)
C20A—C25A	1.344 (11)	N1B—C6B	1.424 (2)
C20A—C21A	1.424 (9)	C1B—C2B	1.383 (2)
C21A—C22A	1.386 (5)	C1B—C6B	1.391 (2)
C21A—H21A	0.9300	C1B—H1BA	0.9300
C22A—C23A	1.371 (6)	C2B—C3B	1.392 (3)
C22A—H22A	0.9300	C2B—H2BA	0.9300
C23A—C24A	1.339 (8)	C3B—C4B	1.376 (3)
C23A—H23A	0.9300	C3B—H3BA	0.9300
C24A—C25A	1.401 (8)	C4B—C5B	1.391 (3)
C24A—H24A	0.9300	C4B—H4BA	0.9300
C25A—H25A	0.9300	C5B—C6B	1.394 (2)
C20C—C21C	1.353 (10)	C5B—H5BA	0.9300
C20C—C25C	1.428 (11)	C7B—C8B	1.429 (2)
C21C—C22C	1.386 (6)	C8B—C9B	1.373 (2)
C21C—H21C	0.9300	C8B—C10B	1.500 (2)
C22C—C23C	1.387 (7)	C9B—C26B	1.489 (2)
C22C—H22C	0.9300	C10B—C17B	1.515 (2)
C23C—C24C	1.388 (7)	C10B—C11B	1.537 (2)
C23C—H23C	0.9300	C10B—H10B	0.9800
C24C—C25C	1.372 (7)	C11B—C12B	1.392 (2)
C24C—H24C	0.9300	C11B—C16B	1.399 (2)
C25C—H25C	0.9300	C12B—C13B	1.386 (2)
C1A—C6A	1.388 (2)	C12B—H12B	0.9300
C1A—C2A	1.390 (2)	C13B—C14B	1.387 (2)
C1A—H1AA	0.9300	C13B—H13B	0.9300
C2A—C3A	1.384 (3)	C14B—C15B	1.388 (2)
C2A—H2AA	0.9300	C14B—H14B	0.9300
C3A—C4A	1.385 (3)	C15B—C16B	1.393 (2)
C3A—H3AA	0.9300	C15B—H15B	0.9300
C4A—C5A	1.388 (2)	C16B—H16B	0.9300
C4A—H4AA	0.9300	C17B—C19B	1.389 (2)
C5A—C6A	1.390 (2)	C17B—C18B	1.405 (2)
C5A—H5AA	0.9300	C18B—C27B	1.498 (2)
C7A—C8A	1.422 (2)	C20B—C21B	1.392 (2)
C8A—C9A	1.376 (2)	C20B—C25B	1.396 (2)
C8A—C10A	1.517 (2)	C21B—C22B	1.389 (2)
C9A—C26A	1.489 (2)	C21B—H21B	0.9300
C10A—C17A	1.512 (2)	C22B—C23B	1.387 (2)
C10A—C11A	1.538 (2)	C22B—H22B	0.9300
C10A—H10A	0.9800	C23B—C24B	1.386 (2)
C11A—C16A	1.392 (2)	C23B—H23B	0.9300
C11A—C12A	1.395 (2)	C24B—C25B	1.386 (2)
C12A—C13A	1.391 (2)	C24B—H24B	0.9300
C12A—H12A	0.9300	C25B—H25B	0.9300
C13A—C14A	1.381 (2)	C26B—H26D	0.9600
C13A—H13A	0.9300	C26B—H26E	0.9600
C14A—C15A	1.381 (3)	C26B—H26F	0.9600
C14A—H14A	0.9300	C27B—H27D	0.9600
C15A—C16A	1.392 (2)	C27B—H27E	0.9600
C15A—H15A	0.9300	C27B—H27F	0.9600
C16A—H16A	0.9300	C28—C29	1.499 (3)
C17A—C19A	1.383 (2)	C28—H28A	0.9600
C17A—C18A	1.412 (2)	C28—H28B	0.9600
C18A—C27A	1.491 (2)	C28—H28C	0.9600
C26A—H26A	0.9600	C29—O3	1.414 (2)
C26A—H26B	0.9600	C29—H29A	0.9700
C26A—H26C	0.9600	C29—H29B	0.9700
C27A—H27A	0.9600	O3—H3	0.8200
			
C19A—O2A—H2OA	109.5	H27A—C27A—H27B	109.5
C7A—N1A—N2A	109.22 (13)	C18A—C27A—H27C	109.5
C7A—N1A—C6A	128.56 (13)	H27A—C27A—H27C	109.5
N2A—N1A—C6A	121.86 (12)	H27B—C27A—H27C	109.5
C9A—N2A—N1A	107.11 (12)	C19B—O2B—H2OB	109.5
C9A—N2A—H2NA	123.4 (15)	C19B—N4B—N3B	110.75 (12)
N1A—N2A—H2NA	119.1 (15)	C19B—N4B—C20B	127.71 (13)
C18A—N3A—N4A	104.78 (13)	N3B—N4B—C20B	119.94 (12)
C19A—N4A—N3A	110.62 (13)	C18B—N3B—N4B	104.91 (12)
C19A—N4A—C20A	131.0 (4)	C9B—N2B—N1B	108.23 (13)
N3A—N4A—C20A	118.0 (4)	C9B—N2B—H2NB	123.5 (13)
C19A—N4A—C20C	126.9 (4)	N1B—N2B—H2NB	119.7 (13)
N3A—N4A—C20C	122.3 (4)	C7B—N1B—N2B	108.79 (13)
C20A—N4A—C20C	4.7 (7)	C7B—N1B—C6B	129.47 (14)
C25A—C20A—N4A	125.2 (7)	N2B—N1B—C6B	120.72 (13)
C25A—C20A—C21A	115.9 (7)	C2B—C1B—C6B	118.92 (17)
N4A—C20A—C21A	118.0 (7)	C2B—C1B—H1BA	120.5
C22A—C21A—C20A	119.9 (5)	C6B—C1B—H1BA	120.5
C22A—C21A—H21A	120.1	C1B—C2B—C3B	120.51 (18)
C20A—C21A—H21A	120.1	C1B—C2B—H2BA	119.7
C23A—C22A—C21A	121.3 (4)	C3B—C2B—H2BA	119.7
C23A—C22A—H22A	119.4	C4B—C3B—C2B	120.17 (18)
C21A—C22A—H22A	119.4	C4B—C3B—H3BA	119.9
C24A—C23A—C22A	118.5 (5)	C2B—C3B—H3BA	119.9
C24A—C23A—H23A	120.7	C3B—C4B—C5B	120.34 (17)
C22A—C23A—H23A	120.7	C3B—C4B—H4BA	119.8
C23A—C24A—C25A	120.7 (5)	C5B—C4B—H4BA	119.8
C23A—C24A—H24A	119.6	C4B—C5B—C6B	119.04 (17)
C25A—C24A—H24A	119.6	C4B—C5B—H5BA	120.5
C20A—C25A—C24A	122.6 (6)	C6B—C5B—H5BA	120.5
C20A—C25A—H25A	118.7	C1B—C6B—C5B	121.02 (16)
C24A—C25A—H25A	118.7	C1B—C6B—N1B	119.51 (15)
C21C—C20C—C25C	120.5 (7)	C5B—C6B—N1B	119.47 (15)
C21C—C20C—N4A	123.6 (7)	O1B—C7B—N1B	123.73 (14)
C25C—C20C—N4A	115.0 (7)	O1B—C7B—C8B	129.79 (15)
C20C—C21C—C22C	120.2 (6)	N1B—C7B—C8B	106.47 (13)
C20C—C21C—H21C	119.9	C9B—C8B—C7B	106.70 (14)
C22C—C21C—H21C	119.9	C9B—C8B—C10B	128.39 (14)
C21C—C22C—C23C	119.9 (4)	C7B—C8B—C10B	124.80 (14)
C21C—C22C—H22C	120.1	N2B—C9B—C8B	109.63 (14)
C23C—C22C—H22C	120.1	N2B—C9B—C26B	119.35 (15)
C22C—C23C—C24C	118.8 (4)	C8B—C9B—C26B	131.00 (15)
C22C—C23C—H23C	120.6	C8B—C10B—C17B	112.66 (12)
C24C—C23C—H23C	120.6	C8B—C10B—C11B	112.68 (12)
C25C—C24C—C23C	122.1 (5)	C17B—C10B—C11B	114.10 (13)
C25C—C24C—H24C	119.0	C8B—C10B—H10B	105.5
C23C—C24C—H24C	119.0	C17B—C10B—H10B	105.5
C24C—C25C—C20C	116.8 (6)	C11B—C10B—H10B	105.5
C24C—C25C—H25C	121.6	C12B—C11B—C16B	118.29 (14)
C20C—C25C—H25C	121.6	C12B—C11B—C10B	119.94 (13)
C6A—C1A—C2A	119.20 (16)	C16B—C11B—C10B	121.63 (14)
C6A—C1A—H1AA	120.4	C13B—C12B—C11B	121.02 (15)
C2A—C1A—H1AA	120.4	C13B—C12B—H12B	119.5
C3A—C2A—C1A	120.86 (16)	C11B—C12B—H12B	119.5
C3A—C2A—H2AA	119.6	C12B—C13B—C14B	120.23 (16)
C1A—C2A—H2AA	119.6	C12B—C13B—H13B	119.9
C2A—C3A—C4A	119.44 (16)	C14B—C13B—H13B	119.9
C2A—C3A—H3AA	120.3	C13B—C14B—C15B	119.71 (15)
C4A—C3A—H3AA	120.3	C13B—C14B—H14B	120.1
C3A—C4A—C5A	120.51 (17)	C15B—C14B—H14B	120.1
C3A—C4A—H4AA	119.7	C14B—C15B—C16B	119.92 (15)
C5A—C4A—H4AA	119.7	C14B—C15B—H15B	120.0
C4A—C5A—C6A	119.54 (16)	C16B—C15B—H15B	120.0
C4A—C5A—H5AA	120.2	C15B—C16B—C11B	120.82 (15)
C6A—C5A—H5AA	120.2	C15B—C16B—H16B	119.6
C1A—C6A—C5A	120.44 (15)	C11B—C16B—H16B	119.6
C1A—C6A—N1A	119.82 (14)	C19B—C17B—C18B	104.75 (13)
C5A—C6A—N1A	119.71 (14)	C19B—C17B—C10B	128.64 (13)
O1A—C7A—N1A	122.85 (14)	C18B—C17B—C10B	126.48 (13)
O1A—C7A—C8A	130.41 (14)	N3B—C18B—C17B	112.30 (13)
N1A—C7A—C8A	106.74 (13)	N3B—C18B—C27B	120.25 (13)
C9A—C8A—C7A	106.64 (13)	C17B—C18B—C27B	127.45 (14)
C9A—C8A—C10A	128.32 (14)	O2B—C19B—N4B	119.10 (13)
C7A—C8A—C10A	124.87 (13)	O2B—C19B—C17B	133.62 (14)
N2A—C9A—C8A	110.07 (14)	N4B—C19B—C17B	107.27 (13)
N2A—C9A—C26A	119.53 (13)	C21B—C20B—C25B	120.29 (14)
C8A—C9A—C26A	130.39 (14)	C21B—C20B—N4B	120.75 (14)
C17A—C10A—C8A	113.93 (12)	C25B—C20B—N4B	118.96 (14)
C17A—C10A—C11A	113.52 (12)	C22B—C21B—C20B	119.19 (15)
C8A—C10A—C11A	111.00 (13)	C22B—C21B—H21B	120.4
C17A—C10A—H10A	105.9	C20B—C21B—H21B	120.4
C8A—C10A—H10A	105.9	C23B—C22B—C21B	120.69 (16)
C11A—C10A—H10A	105.9	C23B—C22B—H22B	119.7
C16A—C11A—C12A	118.28 (14)	C21B—C22B—H22B	119.7
C16A—C11A—C10A	120.60 (14)	C24B—C23B—C22B	119.87 (15)
C12A—C11A—C10A	121.01 (13)	C24B—C23B—H23B	120.1
C13A—C12A—C11A	121.06 (15)	C22B—C23B—H23B	120.1
C13A—C12A—H12A	119.5	C25B—C24B—C23B	120.19 (15)
C11A—C12A—H12A	119.5	C25B—C24B—H24B	119.9
C14A—C13A—C12A	120.13 (16)	C23B—C24B—H24B	119.9
C14A—C13A—H13A	119.9	C24B—C25B—C20B	119.77 (15)
C12A—C13A—H13A	119.9	C24B—C25B—H25B	120.1
C15A—C14A—C13A	119.30 (15)	C20B—C25B—H25B	120.1
C15A—C14A—H14A	120.4	C9B—C26B—H26D	109.5
C13A—C14A—H14A	120.4	C9B—C26B—H26E	109.5
C14A—C15A—C16A	120.93 (16)	H26D—C26B—H26E	109.5
C14A—C15A—H15A	119.5	C9B—C26B—H26F	109.5
C16A—C15A—H15A	119.5	H26D—C26B—H26F	109.5
C15A—C16A—C11A	120.29 (16)	H26E—C26B—H26F	109.5
C15A—C16A—H16A	119.9	C18B—C27B—H27D	109.5
C11A—C16A—H16A	119.9	C18B—C27B—H27E	109.5
C19A—C17A—C18A	104.20 (14)	H27D—C27B—H27E	109.5
C19A—C17A—C10A	128.55 (15)	C18B—C27B—H27F	109.5
C18A—C17A—C10A	127.25 (14)	H27D—C27B—H27F	109.5
N3A—C18A—C17A	112.25 (14)	H27E—C27B—H27F	109.5
N3A—C18A—C27A	120.42 (15)	C29—C28—H28A	109.5
C17A—C18A—C27A	127.33 (15)	C29—C28—H28B	109.5
O2A—C19A—N4A	119.99 (14)	H28A—C28—H28B	109.5
O2A—C19A—C17A	131.81 (15)	C29—C28—H28C	109.5
N4A—C19A—C17A	108.11 (14)	H28A—C28—H28C	109.5
C9A—C26A—H26A	109.5	H28B—C28—H28C	109.5
C9A—C26A—H26B	109.5	O3—C29—C28	113.16 (18)
H26A—C26A—H26B	109.5	O3—C29—H29A	108.9
C9A—C26A—H26C	109.5	C28—C29—H29A	108.9
H26A—C26A—H26C	109.5	O3—C29—H29B	108.9
H26B—C26A—H26C	109.5	C28—C29—H29B	108.9
C18A—C27A—H27A	109.5	H29A—C29—H29B	107.8
C18A—C27A—H27B	109.5	C29—O3—H3	109.5
			
C7A—N1A—N2A—C9A	−4.81 (17)	C20A—N4A—C19A—O2A	12.2 (5)
C6A—N1A—N2A—C9A	−178.43 (14)	C20C—N4A—C19A—O2A	9.1 (5)
C18A—N3A—N4A—C19A	−1.23 (16)	N3A—N4A—C19A—C17A	1.69 (17)
C18A—N3A—N4A—C20A	172.3 (4)	C20A—N4A—C19A—C17A	−170.8 (5)
C18A—N3A—N4A—C20C	174.5 (4)	C20C—N4A—C19A—C17A	−173.8 (5)
C19A—N4A—C20A—C25A	−164.5 (5)	C18A—C17A—C19A—O2A	175.16 (16)
N3A—N4A—C20A—C25A	23.5 (9)	C10A—C17A—C19A—O2A	−4.7 (3)
C20C—N4A—C20A—C25A	−133 (9)	C18A—C17A—C19A—N4A	−1.39 (16)
C19A—N4A—C20A—C21A	26.8 (9)	C10A—C17A—C19A—N4A	178.70 (14)
N3A—N4A—C20A—C21A	−145.2 (5)	C19B—N4B—N3B—C18B	−0.79 (17)
C20C—N4A—C20A—C21A	58 (9)	C20B—N4B—N3B—C18B	−167.49 (14)
C25A—C20A—C21A—C22A	10.8 (9)	C9B—N2B—N1B—C7B	−2.77 (17)
N4A—C20A—C21A—C22A	−179.4 (5)	C9B—N2B—N1B—C6B	−172.31 (13)
C20A—C21A—C22A—C23A	−3.9 (7)	C6B—C1B—C2B—C3B	−0.9 (3)
C21A—C22A—C23A—C24A	−3.9 (7)	C1B—C2B—C3B—C4B	0.1 (3)
C22A—C23A—C24A—C25A	4.5 (8)	C2B—C3B—C4B—C5B	0.4 (3)
N4A—C20A—C25A—C24A	−179.5 (6)	C3B—C4B—C5B—C6B	−0.2 (3)
C21A—C20A—C25A—C24A	−10.5 (10)	C2B—C1B—C6B—C5B	1.2 (3)
C23A—C24A—C25A—C20A	3.1 (9)	C2B—C1B—C6B—N1B	−178.64 (16)
C19A—N4A—C20C—C21C	11.4 (10)	C4B—C5B—C6B—C1B	−0.6 (3)
N3A—N4A—C20C—C21C	−163.6 (6)	C4B—C5B—C6B—N1B	179.18 (16)
C20A—N4A—C20C—C21C	−139 (9)	C7B—N1B—C6B—C1B	29.4 (2)
C19A—N4A—C20C—C25C	−157.5 (4)	N2B—N1B—C6B—C1B	−163.45 (15)
N3A—N4A—C20C—C25C	27.5 (8)	C7B—N1B—C6B—C5B	−150.41 (16)
C20A—N4A—C20C—C25C	52 (9)	N2B—N1B—C6B—C5B	16.7 (2)
C25C—C20C—C21C—C22C	−13.2 (11)	N2B—N1B—C7B—O1B	−178.76 (14)
N4A—C20C—C21C—C22C	178.4 (6)	C6B—N1B—C7B—O1B	−10.4 (2)
C20C—C21C—C22C—C23C	2.4 (9)	N2B—N1B—C7B—C8B	0.17 (16)
C21C—C22C—C23C—C24C	6.4 (8)	C6B—N1B—C7B—C8B	168.50 (14)
C22C—C23C—C24C—C25C	−4.4 (8)	O1B—C7B—C8B—C9B	−178.72 (15)
C23C—C24C—C25C—C20C	−5.9 (8)	N1B—C7B—C8B—C9B	2.43 (16)
C21C—C20C—C25C—C24C	14.9 (11)	O1B—C7B—C8B—C10B	4.9 (2)
N4A—C20C—C25C—C24C	−175.8 (5)	N1B—C7B—C8B—C10B	−173.96 (13)
C6A—C1A—C2A—C3A	0.3 (3)	N1B—N2B—C9B—C8B	4.39 (17)
C1A—C2A—C3A—C4A	−0.1 (3)	N1B—N2B—C9B—C26B	−174.06 (13)
C2A—C3A—C4A—C5A	−0.4 (3)	C7B—C8B—C9B—N2B	−4.22 (17)
C3A—C4A—C5A—C6A	0.7 (3)	C10B—C8B—C9B—N2B	171.99 (14)
C2A—C1A—C6A—C5A	0.0 (2)	C7B—C8B—C9B—C26B	173.99 (16)
C2A—C1A—C6A—N1A	178.35 (14)	C10B—C8B—C9B—C26B	−9.8 (3)
C4A—C5A—C6A—C1A	−0.6 (2)	C9B—C8B—C10B—C17B	109.83 (17)
C4A—C5A—C6A—N1A	−178.87 (15)	C7B—C8B—C10B—C17B	−74.59 (18)
C7A—N1A—C6A—C1A	−47.2 (2)	C9B—C8B—C10B—C11B	−119.33 (17)
N2A—N1A—C6A—C1A	125.08 (16)	C7B—C8B—C10B—C11B	56.26 (18)
C7A—N1A—C6A—C5A	131.11 (17)	C8B—C10B—C11B—C12B	−168.74 (13)
N2A—N1A—C6A—C5A	−56.6 (2)	C17B—C10B—C11B—C12B	−38.62 (19)
N2A—N1A—C7A—O1A	−176.91 (14)	C8B—C10B—C11B—C16B	15.51 (19)
C6A—N1A—C7A—O1A	−3.8 (3)	C17B—C10B—C11B—C16B	145.62 (14)
N2A—N1A—C7A—C8A	3.49 (17)	C16B—C11B—C12B—C13B	0.2 (2)
C6A—N1A—C7A—C8A	176.56 (14)	C10B—C11B—C12B—C13B	−175.73 (14)
O1A—C7A—C8A—C9A	179.56 (16)	C11B—C12B—C13B—C14B	−0.8 (3)
N1A—C7A—C8A—C9A	−0.87 (17)	C12B—C13B—C14B—C15B	0.6 (3)
O1A—C7A—C8A—C10A	−4.8 (3)	C13B—C14B—C15B—C16B	0.2 (2)
N1A—C7A—C8A—C10A	174.72 (14)	C14B—C15B—C16B—C11B	−0.8 (2)
N1A—N2A—C9A—C8A	4.26 (17)	C12B—C11B—C16B—C15B	0.6 (2)
N1A—N2A—C9A—C26A	−176.17 (14)	C10B—C11B—C16B—C15B	176.46 (14)
C7A—C8A—C9A—N2A	−2.12 (17)	C8B—C10B—C17B—C19B	59.2 (2)
C10A—C8A—C9A—N2A	−177.52 (14)	C11B—C10B—C17B—C19B	−70.9 (2)
C7A—C8A—C9A—C26A	178.36 (17)	C8B—C10B—C17B—C18B	−115.95 (17)
C10A—C8A—C9A—C26A	3.0 (3)	C11B—C10B—C17B—C18B	113.92 (17)
C9A—C8A—C10A—C17A	−115.97 (17)	N4B—N3B—C18B—C17B	0.58 (18)
C7A—C8A—C10A—C17A	69.41 (19)	N4B—N3B—C18B—C27B	−179.46 (14)
C9A—C8A—C10A—C11A	114.39 (17)	C19B—C17B—C18B—N3B	−0.16 (19)
C7A—C8A—C10A—C11A	−60.23 (19)	C10B—C17B—C18B—N3B	175.94 (15)
C17A—C10A—C11A—C16A	29.1 (2)	C19B—C17B—C18B—C27B	179.87 (16)
C8A—C10A—C11A—C16A	158.99 (14)	C10B—C17B—C18B—C27B	−4.0 (3)
C17A—C10A—C11A—C12A	−154.73 (15)	N3B—N4B—C19B—O2B	−179.02 (13)
C8A—C10A—C11A—C12A	−24.9 (2)	C20B—N4B—C19B—O2B	−13.6 (2)
C16A—C11A—C12A—C13A	−0.7 (2)	N3B—N4B—C19B—C17B	0.71 (18)
C10A—C11A—C12A—C13A	−176.92 (15)	C20B—N4B—C19B—C17B	166.11 (15)
C11A—C12A—C13A—C14A	0.7 (3)	C18B—C17B—C19B—O2B	179.34 (17)
C12A—C13A—C14A—C15A	0.1 (3)	C10B—C17B—C19B—O2B	3.4 (3)
C13A—C14A—C15A—C16A	−0.9 (3)	C18B—C17B—C19B—N4B	−0.33 (17)
C14A—C15A—C16A—C11A	0.9 (3)	C10B—C17B—C19B—N4B	−176.32 (15)
C12A—C11A—C16A—C15A	−0.1 (2)	C19B—N4B—C20B—C21B	48.7 (2)
C10A—C11A—C16A—C15A	176.12 (16)	N3B—N4B—C20B—C21B	−147.13 (15)
C8A—C10A—C17A—C19A	−69.5 (2)	C19B—N4B—C20B—C25B	−130.94 (17)
C11A—C10A—C17A—C19A	58.8 (2)	N3B—N4B—C20B—C25B	33.3 (2)
C8A—C10A—C17A—C18A	110.58 (17)	C25B—C20B—C21B—C22B	0.6 (2)
C11A—C10A—C17A—C18A	−121.05 (16)	N4B—C20B—C21B—C22B	−178.97 (15)
N4A—N3A—C18A—C17A	0.32 (17)	C20B—C21B—C22B—C23B	−0.1 (3)
N4A—N3A—C18A—C27A	−179.89 (14)	C21B—C22B—C23B—C24B	−0.3 (3)
C19A—C17A—C18A—N3A	0.66 (17)	C22B—C23B—C24B—C25B	0.2 (3)
C10A—C17A—C18A—N3A	−179.42 (14)	C23B—C24B—C25B—C20B	0.3 (3)
C19A—C17A—C18A—C27A	−179.11 (16)	C21B—C20B—C25B—C24B	−0.7 (2)
C10A—C17A—C18A—C27A	0.8 (3)	N4B—C20B—C25B—C24B	178.90 (15)
N3A—N4A—C19A—O2A	−175.35 (13)		

### Hydrogen-bond geometry (Å, °)

**Table:**

*D*—H···*A*	*D*—H	H···*A*	*D*···*A*	*D*—H···*A*
O2A—H2OA···O1A	0.82	1.76	2.5755 (16)	173
O3—H3···N3A^i^	0.82	2.11	2.923 (2)	173
O2B—H2OB···O1B	0.82	1.68	2.4970 (16)	176
N2A—H2NA···N3B^ii^	0.93 (3)	1.88 (3)	2.810 (2)	176 (2)
N2B—H2NB···O1A^iii^	0.95 (2)	1.75 (2)	2.6922 (19)	173 (2)
C1B—H1BA···O1B	0.93	2.37	2.925 (2)	118
C2B—H2BA···O3^iv^	0.93	2.48	3.313 (2)	149
C21A—H21A···O2A	0.93	2.41	2.910 (5)	114
C5B—H5BA···Cg1^i^	0.93	2.89	3.595 (3)	134
C29—H29B···Cg2^iv^	0.97	2.80	3.492 (2)	129

Symmetry codes: (i) −*x*+1, −*y*+1, −*z*+1; (ii) −*x*+1, −*y*+1, −*z*; (iii) *x*+1, *y*+1, *z*; (iv) *x*+1, *y*, *z*.
